# Digital Health Platform for Maternal Health: Design, Recruitment Strategies, and Lessons Learned From the PowerMom Observational Cohort Study

**DOI:** 10.2196/70149

**Published:** 2025-04-07

**Authors:** Toluwalase Ajayi, Jacqueline Kueper, Lauren Ariniello, Diana Ho, Felipe Delgado, Matthew Beal, Jill Waalen, Katie Baca Motes, Edward Ramos

**Affiliations:** 1 Jacobs Center for Health Innovation Department of Medicine and Pediatrics University of California, San Diego La Jolla, CA United States; 2 Digital Trial Center Scripps Research Translational Institute La Jolla, CA United States; 3 Division of Preventative Medicine Department of Family Medicine University of California, San Diego La Jolla United States

**Keywords:** maternal health research, digital health platforms, pregnancy monitoring, decentralized clinical trials, participant engagement, health disparities

## Abstract

**Background:**

Maternal health research faces challenges in participant recruitment, retention, and data collection, particularly among underrepresented populations. Digital health platforms like PowerMom (Scripps Research) offer scalable solutions, enabling decentralized, real-world data collection. Using innovative recruitment and multimodal techniques, PowerMom engages diverse cohorts to gather longitudinal and episodic data during pregnancy and post partum.

**Objective:**

This study aimed to evaluate the design, implementation, and outcomes of the PowerMom research platform, with a focus on participant recruitment, engagement, and data collection across diverse populations. Secondary objectives included identifying challenges encountered during implementation and deriving lessons to inform future digital maternal health studies.

**Methods:**

Participants were recruited via digital advertisements, pregnancy apps, and the PowerMom Consortium of more than 15 local and national organizations. Data collection included self-reported surveys, wearable devices, and electronic health records. Anomaly detection measures were implemented to address fraudulent enrollment activity. Recruitment trends and descriptive statistics from survey data were analyzed to summarize participant characteristics, assess engagement metrics, and quantify missing data to identify gaps.

**Results:**

Overall, 5617 participants were enrolled from 2021 to 2024, with 69.8% (n=3922) providing demographic data. Of these, 48.5% (2723/5617) were younger than 35 years, 14% (788/5617) identified as Hispanic or Latina, and 13.7% (770/5617) identified as Black or African American. Geographic representation spanned all 50 US states, Puerto Rico, and Guam, with 58.3% (3276/5617) residing in areas with moderate access to maternity care and 16.4% (919/5617) in highly disadvantaged neighborhoods based on the Area Deprivation Index. Enrollment rates increased substantially over the study period, from 55 participants in late 2021 to 3310 in 2024, averaging 99.4 enrollments per week in 2024. Participants completed a total of 17,123 surveys, with 71.8% (4033/5617) completing the Intake Survey and 12.4% (697/5617) completing the Postpartum Survey. Wearable device data were shared by 1168 participants, providing more than 378,000 daily biometric measurements, including activity levels, sleep, and heart rate. Additionally, 96 participants connected their electronic health records, contributing 276 data points such as diagnoses, medications, and laboratory results. Among pregnancy-related characteristics, 28.1% (1578/5617) enrolled during the first trimester, while 15.1% (849/5617) reported information about the completion of their pregnancies during the study period. Among the 913 participants who shared delivery information, 56.1% (n=512) had spontaneous vaginal deliveries and 17.9% (n=163) underwent unplanned cesarean sections.

**Conclusions:**

The PowerMom platform demonstrates the feasibility of using digital tools to recruit and engage diverse populations in maternal health research. Its ability to integrate multimodal data sources showcases its potential to provide comprehensive maternal-fetal health insights. Challenges with data completeness and survey attrition underscore the need for sustained participant engagement strategies. These findings offer valuable lessons for scaling digital health platforms and addressing disparities in maternal health research.

**Trial Registration:**

ClinicalTrials.gov NCT03085875; https://clinicaltrials.gov/study/NCT03085875

## Introduction

In 2020, there were approximately 24 pregnancy-related deaths per 100,000 births in the United States, which is more than double compared with other high-income countries [1]. Alarmingly, these rates have continued to rise, with disparities disproportionately affecting marginalized populations [2]. Non-Hispanic Black or African American pregnant individuals experience maternal mortality rates approximately 2.5 times higher than their White counterparts, with non-Hispanic American Indian or Alaska Native women facing rates three times higher [2,3]. Cardiovascular conditions, perinatal depression, and preexisting medical conditions are among the leading contributors to these disparities [4,5]. However, the root causes extend beyond medical risk factors, with social determinants of health and structural racism playing crucial roles [6,7].

Access to care, socioeconomic status, implicit bias, and environmental factors further exacerbate these inequities. Individuals from underrepresented communities are more likely to face barriers to health care, economic insecurity, and a lack of culturally competent care. These disparities are often intensified by distrust of health care systems, fueled by historical and ongoing discrimination in medical settings [8]. Addressing these disparities requires innovative research approaches that not only engage affected communities but also prioritize trust, accessibility, and cultural competence [9].

Traditional maternal health research has struggled to effectively recruit and engage underrepresented populations. Common recruitment barriers include mistrust of the health care system, concerns about data confidentiality, language differences, and logistical hurdles such as the lack of transportation or time constraints [10,11]. These challenges are compounded by the fact that traditional recruitment methods, such as clinic-based enrollment, often fail to reach the most vulnerable populations, leading to underrepresentation in research that is critical for health equity [12,13]. However, many of these traditional methods do not offer sufficient direct benefits to participants to offset their perceived and real costs of participation, particularly in communities with heightened mistrust [9].

The emergence of digital health platforms offers a potential solution to overcome these long-standing barriers, particularly in maternal health research [14]. Mobile and decentralized platforms provide an opportunity to recruit participants remotely, allowing for broad geographical reach and the ability to engage individuals in real-world settings [15]. Digital tools enable continuous, real-time data collection, which can provide a more holistic view of maternal health than episodic clinic visits. These platforms can also deliver tailored educational content and resources, building trust and empowering participants through transparency and engagement [16].

The PowerMom (Scripps Research) platform was designed to capitalize on these opportunities by implementing a bilingual (English and Spanish) digital research platform that facilitates remote, convenient participation. PowerMom’s recruitment strategy was explicitly designed to harness the power of digital platforms to allow seamless, rolling self-enrollment and ensure wide-scale and inclusive participation across diverse communities. It leverages digital approaches such as push notifications and messaging to enhance participant engagement and retention [17-20], allowing for data collection that respects participants’ time and cultural needs.

By providing a decentralized clinical trial environment, PowerMom enables participants to engage with the study on their own terms, enhancing adherence and yielding data that reflect a broad, comprehensive, and participant-centered perspective on how structural factors influence maternal health. The platform supports the collection of real-world, continuous data through app-based surveys, electronic health record (EHR) data linkage, and digital health technologies such as wearable sensors, capturing biometric data (eg, heart rate, sleep, and physical activity) that go beyond the traditional, episodic measures collected during prenatal clinic visits [21-26]. PowerMom’s flexible architecture supports not only the collection of baseline data but also the integration of multiple substudies, which allows for more targeted research on specific maternal health issues while leveraging the broader cohort’s data.

In this paper, we describe the design and methods of the PowerMom research platform, focusing on its recruitment strategies, data collection capabilities, and participant engagement efforts, and discuss lessons learned from its implementation in diverse populations. In addition, we provide a detailed description of the participant demographics recruited through PowerMom and examine their engagement with the various data collection opportunities throughout the pregnancy and postpartum stages.

## Methods

### Platform Design

The PowerMom platform, launched in March 2021, is built on CareEvolution’s MyDataHelps secure digital infrastructure, which provides a flexible and scalable foundation for collecting and integrating a range of participant data, including biometric, survey, and EHR data. This cloud-based, Health Insurance Portability and Accountability Act (HIPAA)–compliant infrastructure provides secure data storage and management, leveraging CareEvolution’s backend architecture to maintain participant confidentiality and data integrity. In addition, the platform’s technical infrastructure adheres to industry-leading security protocols for data transfer, ensuring that biometric data from wearables, EHR data, and survey responses are securely encrypted and processed.

The PowerMom platform integrates data from various sources in real time, allowing for continuous monitoring of participants’ health. This includes heart rate, activity levels, and sleep data collected from wearable devices such as Fitbit, Apple Watch, and other HealthKit or Google Fit–compatible technologies. By allowing participants to connect these devices, the platform facilitates a richer, more granular view of maternal health, capturing data beyond the traditional measures obtained during prenatal clinic visits.

To ensure that the platform reflects the needs of its target population, PowerMom’s development involved collaboration with a participant advisory board composed of pregnant and postpartum individuals from underrepresented communities. The advisory board provided critical feedback on the platform’s design, user interface, and engagement strategies, ensuring that the platform remains culturally relevant and user-friendly for diverse participants. This adheres to the CONSORT-EHEALTH (v1.6.1) guidelines for digital health intervention research to ensure comprehensive and standardized reporting. The checklist was used to structure study reporting, covering recruitment, platform usability, data integration, and participant engagement.

### Participant Recruitment

#### Eligibility Criteria

Participants were eligible for PowerMom if they were pregnant or post partum (up to 8 weeks after delivery), aged 16 years and older, residing in the United States or its territories, and capable of providing consent in either English or Spanish.

#### eConsent

All eligible PowerMom participants were presented with an innovative electronic consent (eConsent) process, enabling them to remotely join the study. This eConsent process significantly broadened the potential participant pool by allowing individuals to complete consent forms at their convenience, reducing the need for in-person visits or transfer of paper forms. The eConsent interface was designed to be user-friendly and accessible across different devices, ensuring that participants could easily navigate the process (refer to Multimedia Appendix 1). This digital consent process not only facilitated seamless participation but also ensured compliance with ethical standards for informed consent in clinical research.

#### Study Setting

The study was conducted entirely remotely, using the PowerMom platform to engage participants across the United States, Puerto Rico, and Guam. The decentralized nature of the study eliminated geographic barriers, enabling recruitment from urban, suburban, and rural areas. Participants could enroll and participate using mobile devices or computers, providing flexibility for those in resource-limited settings.

#### Recruitment Strategy

PowerMom implemented a rolling, digital self-enrollment process to maximize reach and engagement. Recruitment took place from March 22, 2021, to July 30, 2024, and relied on a variety of digital and in-person outreach strategies. Participants in PowerMom are recruited into a baseline cohort but may also be invited to join additional substudies based on specific eligibility criteria. These substudies focus on more targeted research questions, allowing researchers to augment baseline data with additional detailed outcomes.

#### Digital Outreach

Recruitment relied heavily on digital advertising campaigns on social media platforms such as Facebook, Instagram, and Google Ads. In addition, partnerships with pregnancy-related apps such as Philips Pregnancy+ (Philips Digital) enabled in-app advertisements that reached individuals in their natural digital environments. These ads provided information about the study, emphasizing its accessibility and participant-centered design. Digital enrollment campaigns were designed to appear within the app, enabling participants to enroll in PowerMom seamlessly from their daily digital environment.

#### PowerMom Consortium

The PowerMom Consortium, established in December 2021, brought together more than 15 local and national organizations focused on improving maternal health. This consortium amplified recruitment efforts through joint campaigns, newsletters, and educational outreach, significantly increasing visibility among target demographics [27].

#### Community Engagement

One of the platform’s key partners, Mae Health, is a culturally competent, digital health platform that connects Black expectant mothers with vital resources to improve pregnancy outcomes. The collaboration with Mae Health included educational campaigns and targeted outreach to Black and African American communities.

PowerMom also partnered with advocacy organizations such as the African American Wellness Center for Children and Families. These organizations provided on-the-ground recruitment efforts, ensuring that PowerMom reached communities that might otherwise be underrepresented in digital health research.

### Data Collection

#### Self-Reported Outcomes

Participants were asked to complete several surveys throughout the study. An initial Intake Survey gathered demographic data, including race, ethnicity, education, socioeconomic status, and health care access. A Health History Survey collected information on pregnancy history, preexisting health conditions, and access to care.

Following the baseline surveys, participants received biweekly surveys to assess changes in their health during pregnancy. These surveys collected data on vital signs (eg, weight, blood pressure, and heart rate); pregnancy symptoms; and health behaviors, including tobacco, alcohol, and drug use. Biweekly surveys were released based on the completion of the Intake and Health History Surveys, and participants were notified about each new biweekly survey release via push notifications through the PowerMom app.

Participants also completed a Delivery Survey upon giving birth, capturing details about the delivery, birth outcomes, and complications. A Postpartum Survey, administered at 6-8 weeks post partum, assessed maternal mental health using the Edinburgh Postnatal Depression Scale and gathered information on postpartum complications such as gestational diabetes, pregnancy-induced hypertension, preeclampsia, and preterm birth (refer to Multimedia Appendix 1).

#### Wearable Devices

Participants were prompted to connect their wearable devices, including Fitbit, Apple Watch, and other compatible devices, for real-time monitoring of heart rate, physical activity, and sleep duration data. Participants had the option to upload up to 3 months of retrospective data (eg, prepregnancy data) and continue data collection prospectively throughout their pregnancy and the postpartum period.

#### EHR Integration

After completing the initial baseline surveys, participants were prompted to connect their pregnancy-related EHR data through the PowerMom app. The EHR integration can reach more than 85% of all US health care providers, ensuring comprehensive coverage of both historical and prospective health data. Participants could connect their EHR at any point during the study, providing a continuous flow of clinical information that complemented the self-reported and biometric data.

### Study Engagement and Retention Strategies

The PowerMom platform was designed with a strong focus on participant engagement and retention. To keep participants engaged, the platform provided regular push notifications, educational content, and personalized data visualizations. For example, participants could track their own metrics—such as daily activity or sleep patterns—through visual feedback in the app, which helped build trust and maintain engagement.

In addition to digital strategies, participants were engaged through blog posts that provided some initial return of results based on aggregated data collected. The PowerMom advisory board also contributed to refining engagement strategies by providing input on how to make the platform more user-friendly and responsive to the needs of underrepresented populations. Engagement metrics were closely monitored, and iterative improvements were made to the platform based on participant feedback.

### Data Integration

All data types (eg, survey data, wearable device data, and EHR data) were collected and integrated through the MyDataHelps PowerMom platform app. When discrepancies arose between data sources, such as activity metrics reported in surveys versus wearables, priority was given to wearable data due to their objective and continuous measurement capabilities. Survey data were used to provide context, particularly when participants reported conditions or limitations that could explain deviations. This approach ensured the accuracy and reliability of the data.

These data were securely transferred to the Scripps Research Institute for storage and analysis. The data management system leverages cloud-based infrastructure that is HIPAA compliant, ensuring the highest level of data integrity and security. The data were regularly monitored by the research team and cleaned to address any issues with synchronization between devices, missing data, or outliers, ensuring high-quality data for analysis.

### Statistical Analysis

We evaluated the PowerMom platform using operational outcomes and participant characteristics. The analytic sample included all participants who met the baseline PowerMom eligibility criteria, assessed through an initial Screener Survey, and who provided eConsent to participation.

### Participant Characteristics

Baseline sociodemographic and pregnancy-related characteristics of all enrolled participants were summarized using self-report data from the Screener, Intake, and Health History Surveys. Delivery outcomes were also described for participants who filled out the Delivery Survey. All eligible participants are included in table summaries of these characteristics, with missing data represented as “Unknown” responses for both single-question and survey-level nonresponse. Survey-level completion patterns, reported in the engagement metrics section, capture the number of eligible participants who completed at least one question on each survey.

### Enrollment Trends

We analyzed enrollment trends beyond the three-year study recruitment period. Weekly and cumulative enrollment rates were calculated to demonstrate the impact of these strategies, culminating in the final total number of participants.

Summary statistics were calculated using frequencies and percentages for categorical variables and mean (SD) for continuous variables. All analyses were conducted using MyDataHelps Export Explorer (SQL) and Python (v3.8; Guido van Rossum).

### Ethical Considerations

The study protocol for PowerMom was reviewed and approved by the Scripps Health Institutional Review Board (IRB 21-7738). The research adhered to ethical guidelines for human subject research, ensuring compliance with all applicable regulations. All participants provided eConsent before enrollment. The eConsent process was designed to be accessible and user-friendly, enabling participants to complete the consent remotely via digital platforms. The consent process highlighted participants’ rights, data confidentiality measures, and the voluntary nature of their participation. Participant data were anonymized or deidentified before analysis to ensure confidentiality. The PowerMom platform operates on a HIPAA-compliant, secure infrastructure, with additional protections provided by a Certificate of Confidentiality from the National Institutes of Health. This legal protection allows the platform to refuse the disclosure of participant data in civil, criminal, administrative, or legislative proceedings, adding an extra robust layer of privacy beyond standard HIPAA protections. Participants were not compensated for being part of the PowerMom study. However, participants recruited from the PowerMom study for one substudy were provided with monetary incentives. Participants in this substudy were eligible to receive up to US $175 in Amazon gift cards as compensation for completing specific milestones within the substudy. These incentives were structured to encourage engagement without coercion, according to institutional review board guidelines, and the details were explicitly communicated during the consent process.

## Results

### Demographic Data

Participants were drawn from diverse demographic backgrounds, with representation across different age groups, racial and ethnic categories, and geographic locations. Among the 5617 participants enrolled in the study, 3922 (69.8%) opted to share demographic information. Participants younger than 35 years comprised 48.5% (2723/5617) of the cohort, while 19.3% (1083/5617) were 35 years or older. Age data were missing for 32.2% (1811/5617) of participants.

Regarding ethnicity, 14% (788/5617) of participants reported identifying as Hispanic or Latina, while 55.8% (3134/5617) did not. Ethnicity data were missing for 30.2% (1695/5617) of participants. Racial backgrounds included 0.9% (52/5617) identifying as American Indian or Alaska Native, 1.9% (105/5617) as Asian, 13.7% (770/5617) as Black or African American or African, and 38.7% (2175/5617) as White. In addition, 8.5% (478/5617) identified as Hispanic, Latina, or Spanish without specifying another race, 0.2% (9/5617) as Native Hawaiian or Other Pacific Islander, and 5.9% (333/5617) as multiple races. Racial data were missing for 30.2% (1695/5617) of participants.

Geographic distribution based on zip code at the time of study enrollment showed that 11.3% (635/5617) of participants residing in the Northeast, 29% (1630/5617) in the South, 15.7% (880/5617) in the Midwest, and 15.2% (853/5617) in the West. Only 0.4% (22/5617) resided in the Pacific region, and less than 0.1% (6/5617) were from Puerto Rico. Geographic data were missing for 28.4% (1594/5617) of participants.

Education levels varied, with 12.5% (703/5617) of participants reporting a graduate or postgraduate degree, 18.9% (1063/5617) holding a college degree, and 6.1% (342/5617) reporting some college or technical school education. High-school graduates or those with General Educational Development attainment accounted for 27.5% (1545/5617), while 6% (337/5617) reported not completed high school. Education data were missing for 29% (1627/5617) of participants.

Access to maternity care was categorized based on geographic location using the March of Dimes Maternity Care Deserts Dashboard [28]. These data were reported by 62.6% (3520/5617) of participants, with 58.3% (3276/5617) residing in areas with moderate access and 4.3% (244/5617) in maternity care deserts or areas with low access. Access data were missing for 37.3% (2097/5617) of participants.

Neighborhood disadvantage was assessed using the Neighborhood Atlas’ Area Deprivation Index (ADI). The higher the ADI value, the less resources the neighborhood has. Among all participants, 16.4% (919/5617) reported residence in neighborhoods with an ADI national rank of greater than or equal to 75, indicating higher levels of deprivation, while 24.6% (1381/5617), 19.7% (1108/5617), and 10.2% (574/5617) lived in areas with ADI national ranks between 50-75, 25-50, and less than 25, respectively. ADI data were missing for 29.1% (1635/5617) of participants.

### Pregnancy and Delivery-Related Characteristics

Among the 5617 participants enrolled in the study, the pregnancy stage at enrollment varied. Approximately 28.1% (1578/5617) of participants enrolled during the first trimester, 26.6% (1493/5617) in the second trimester, and 16.2% (909/5617) in the third trimester. Postdelivery enrollment accounted for 13.1% (736/5617), while the pregnancy stage at enrollment was unknown for 16% (901/5617) of participants.

Most participants (3394/5617, 60.4%) reported being pregnant with one fetus, while 2.2% (121/5617) reported being pregnant with twins, 0.3% (16/5617) with triplets, and 0.3% (15/5617) with more than 3 fetuses. According to available data [29], around 2% to 3% of pregnancies in the United States result in the birth of triplets or higher-order multiples. The number of fetuses was not reported for 36.9% (2071/5617) of participants.

Regarding pregnancy history, 6.6% (371/5617) of participants reported no previous pregnancies, while 13.5% (759/5617), 12.2% (688/5617), 8% (447/5617), 5.8% (328/5617), and 3.2% (180/5617) reported 1, 2, 3, 4, and 5 previous pregnancies, respectively. In addition, 4.7% (266/5617) of participants reported more than 5 previous pregnancies, and 45.9% (2578/5617) did not provide information on past pregnancies.

When asked about the number of past births, 19.9% (1118/5617) reported having none, while 16.4% (923/5617), 10.2% (571/5617), 4.6% (260/5617), 2% (111/5617, 1% (58/5617), and 0.9% (53/5617) reported 1, 2, 3, 4, 5, and more than 5 births, respectively. Information on past births was missing for 44.9% (2523/5617) of participants. Of the 1106 (21.1%) participants who reported at least one previous pregnancy and a complication, the most commonly reported pregnancy complications were miscarriage (n=637), preterm birth (n=250), and high blood pressure (n=234).

Participants were seeing a variety of prenatal care providers at baseline, with 37.5% (2104/5617) reported seeing a physician; 9.9% (555/5617) seeing a midwife; 2.3% (129/5617) seeing a doula; and smaller proportions seeing other providers such as nurse midwives (56/5617, 1%) or nurse practitioners (36/5617, 0.6%) or planning home births (9/5617, 0.2%). However, 8% (447/5617) reported not seeing any prenatal care provider, and 44.7% (2511/5617) did not report their provider.

Mental health treatment at baseline revealed that 3.4% (189/5617) of participants were being treated for anxiety, 1.8% (102/5617) for depression, and 9.8% (550/5617) for both anxiety and depression, while 40.9% (2297/5617) reported receiving no treatment. This information was missing for 44.2% (2480/5617) of participants. In addition, 51.4% (2889/5617) of participants reported taking medications or supplements at baseline, 4.6% (261/5617) reported taking none, and 44% (2469/5617) did not report this information. Among the 1449 (25.8%) participants who reported at least one prepregnancy condition, the three most common were COVID-19 (n=781), endometriosis (n=316), and a sexually transmitted illness (n=217).

Regarding family medical history, 9.2% (517/5617) of participants reported having a family member diagnosed with conditions or diseases during childhood, while 46.3% (2603/5617) did not, and 44.5% (2497/5617) did not provide this information. Of the 9.2% (517/5617) of participants that reported themselves, the baby’s second biological parent, or anyone in their family having ever had any conditions or diseases diagnosed when they were a baby or in early childhood, the three most common diagnoses were heart defect (n=83), chromosome genetic disorder (n=58), and sickle cell disease or trait (n=21).

For delivery outcomes, 15.1% (849/5617) of participants reported giving birth, 0.9% (52/5617) reported a pregnancy loss, and 0.2% (12/5617) reported stillbirths. Delivery type information showed that 9.1% (512//5617) reported having spontaneous vaginal deliveries, 2.9% (163/5617) had unplanned cesarean sections, and 2.1% (117/5617) had planned cesarean sections. Forceps deliveries were reported by 0.1% (6/5617), vacuum extractions by 0.4% (20/5617), and vaginal births after cesarean by 0.4% (27/5617). Delivery type was not reported by 83.8% (4709/5617) of participants.

Finally, 14.4% (807/5617) of participants reported delivering in a hospital, 0.4% (22/5617) at home, and 0.3% (18/5617) in birthing centers. Delivery location data was unknown for 84.9% (4768/5617) of participants.

### Engagement Trends and Survey Completion

The PowerMom platform collected a total of 17,123 individual surveys from 5617 pregnant and postpartum participants across all 50 US states, Puerto Rico, and Guam. Of the 5617 participants who completed the initial Screener Survey, 4031 (71.76%) completed the Intake Survey and 3148 (56.04%) completed the Health History Survey, which were the two baseline surveys administered. There were 1514 (26.95%) participants who completed at least one biweekly Health and Wellbeing Survey; 913 (16.25%) who completed the Delivery Survey; and 697 (12.41%) who completed the Postpartum Survey, which assessed mental health and was sent 6-8 weeks after the participant’s reported delivery date.

### Fraudulent Behavior

Fraud detection was an essential part of maintaining data integrity. Metrics such as unusually short completion times for eConsent or surveys, duplicate IP addresses, and repetitive data patterns were flagged. 580 of the flagged cases were determined to be fraud and excluded.

### Enrollment Trends

Enrollment rates varied significantly from the platform’s launch in 2021 through 2024. In the first year of PowerMom’s launch, 70 participants were enrolled, with an average of 2.6 participants per week. As recruitment methods were refined and partnerships with pregnancy apps such as Philips Pregnancy+ and Mae Health were formed, the average enrollment rate increased substantially, reaching an average of 93 participants per week by 2024. Table 1 shows the enrollment trends by year, with a clear rise in participant engagement.

**Table 1:** Participant enrollment rates for the PowerMom study over time.

Year	Total participants (per year), n	Rate (per month)	Rate (per week)
2021 (starting on September 15, 2021)	55	15.7	3.6
2022	481	40.1	9.3
2023	1771	147.6	34.1
2024 (ending on August 22, 2024)	3310	427.1	99.4

### Wearable Device Data

Of the 5617 participants, 1168 (20.8%) elected to share data from their wearable devices. The distribution of device use is as follows: 54.1% (632/1168) from Fitbit, 40.6% (474/1168) from Apple HealthKit, and 5.3% (62/1168 participants) from Google Fit. A total of 378,624 daily measurements were collected from participants, covering activity levels, sleep duration, and resting heart rate. These real-time biometric data points provide a detailed, continuous view of participants’ health throughout their pregnancy journey.

### EHR Data

Connecting EHR data was optional for PowerMom participants. A total of 96 (1.7%) participants chose to connect their EHR with the PowerMom platform, providing 276 distinct data points. These data points included diagnosis codes, lab results, medications, and procedures.

Table 2 presents the summary of the demographic, geographic, and access-to-care data for 5617 participants enrolled in the PowerMom study (2021-2024). The table includes age distribution, Hispanic or Latina identification, racial background, education levels, geographic regions (eg, Northeast and Midwest), and levels of access to maternity care based on the March of Dimes Maternity Care Deserts Dashboard. Data also include neighborhood deprivation as assessed by the ADI. Missing data are reported for each category.

Table 3 presents a summary of baseline health history and pregnancy-related characteristics for 5617 participants enrolled in the PowerMom study. The table includes pregnancy stage at enrollment, number of fetuses, previous pregnancies and births, prenatal care providers, and medication or supplement usage at baseline. In addition, the table details conditions such as prepregnancy and past pregnancy complications, mental health treatment (anxiety and depression), and family disease history. Delivery outcomes, types, and locations are also reported. Missing or unknown data percentages are indicated for each category to account for variability in reporting.

Table 1 presents participant enrollment rates for the PowerMom study, displayed by year. The table reports the total number of participants enrolled each year, along with calculated monthly and weekly enrollment rates. These trends highlight substantial increases in recruitment efficiency, particularly in 2023 and 2024, reflecting the impact of enhanced outreach strategies and platform optimization.

**Table 2:** Demographic and regional characteristics of PowerMom participants.

Characteristics			Values (N=5617), n (%)		
**Age category (years)**					
	<35	2723 (48.5)			
	≥35	1083 (19.3)			
	Missing	1811 (32.2)			
**Hispanic or Latina**					
	Yes	788 (14.0)			
	No	3134 (55.8)			
	Missing	1695 (30.2)			
**Racial background**					
	American Indian or Alaska Native	52 (0.9)			
	Asian	105 (1.9)			
	Black or African American or African	770 (13.7)			
	Hispanic, Latina, or Spanish (no other race and ethnicity specified)	478 (8.5)			
	Native Hawaiian or Other Pacific Islander	9 (0.2)			
	White	2175 (38.7)			
	Multiple races	333 (5.9)			
	Missing	1695 (30.2)			
**Region**					
	Northeast	635 (11.3)			
	South	1630 (29.0)			
	Midwest	880 (15.7)			
	West	853 (15.2)			
	Pacific	24 (0.4)			
	Puerto Rico	1 (<0.1)			
	Missing	1594 (28.4)			
**Education**					
	Graduate or postgraduate degree	703 (12.5)			
	College degree	1063 (18.9)			
	Some college or technical school	342 (6.1)			
	High school graduate or GED^a^ (grade 12)	1545 (27.5)			
	Some high school (grades 9-11)	337 (6.0)			
	Missing	1627 (29.0)			
**Level of access to maternity care**					
	Moderate access to care/access to maternity care	3276 (58.3)			
	Maternity care desert/low access to care	244 (4.3)			
	Missing	2097 (37.3)			
**Neighborhood Atlas’s ADI^b^**					
	≥75	919 (16.4)			
	≥50 and <75	1381 (24.6)			
	≥25 and <50	1108 (19.7)			
	<25	574 (10.2)			
	Missing	1635 (29.1)			

^a^GED: General Educational Development.

^b^ADI: Area Deprivation Index.

**Table 3:** Health history and pregnancy characteristics of PowerMom participants.

Characteristics		Values (N=5617), n (%)
**Pregnancy stage at enrollment**		
	First trimester	1578 (28.1)
	Second trimester	1493 (26.6)
	Third trimester	909 (16.2)
	Post delivery	736 (13.1)
	Unknown	901 (16.0)
**Number of fetuses**		
	1	3394 (60.4)
	2	121 (2.2)
	3	16 (0.3)
	>3	15 (0.3)
	Unknown	2071 (36.9)
**Number of past pregnancies**		
	0	371 (6.6)
	1	759 (13.5)
	2	688 (12.2)
	3	447 (8.0)
	4	328 (5.8)
	5	180 (3.2)
	>5	266 (4.7)
	Unknown	2578 (45.9)
**Number of past births**		
	0	1118 (19.9)
	1	923 (16.4)
	2	571 (10.2)
	3	260 (4.6)
	4	111 (2)
	5	58 (1)
	>5	53 (0.9)
	Unknown	2523 (44.9)
**Prenatal care providers seen at baseline**		
	Doula	129 (2.3)
	Homebirth	9 (0.2)
	Physician	2104 (37.5)
	Midwife	555 (9.9)
	Nurse midwife	56 (1)
	Nurse practitioner	36 (0.6)
	Obstetrician	237 (4.2)
	Other	143 (2.5)
	None	447 (8.0)
	Unknown	2511 (44.7)
**Taking medications or supplements at baseline**		
	No	261 (4.6)
	Yes	2887 (51.4)
	Unknown	2469 (44.0)
**Receiving anxiety or depression treatment at baseline**		
	Anxiety and depression	550 (9.8)
	Anxiety only	189 (3.4)
	Depression only	102 (1.8)
	Neither	2296 (40.9)
	Unknown	2480 (44.2)
**Parent or familial childhood condition or disease history**		
	No	2603 (46.3)
	Yes	517 (9.2)
	Unknown	2497 (44.5)
**Prepregnancy condition**		
	No	1699 (30.2)
	Yes	1449 (25.8)
	Unknown	2469 (44.0)
**Past pregnancy complication**		
	No	1575 (28.0)
	Yes	1106 (19.7)
	Not applicable	371 (6.6)
	Unknown	2565 (45.7)
**Pregnancy completion**		
	Gave birth	849 (15.1)
	Pregnancy ended	52 (0.9)
	Stillbirth	12 (0.2)
	Unknown	4704 (83.7)
**Delivery type**		
	Forceps delivery	5 (0.1)
	Not applicable	64 (1.1)
	Planned cesarean section	117 (2.1)
	Spontaneous vaginal delivery	512 (9.1)
	Unknown	4709 (83.8)
	Unplanned C-section	163 (2.9)
	VBAC^a^	27 (0.5)
	Vacuum extraction	20 (0.4)
**Delivery location**		
	Birthing center	18 (0.3)
	Home	22 (0.4)
	Hospital	807 (14.4)
	Other	2 (0)
	Unknown	4768 (84.9)

^a^VBAC: vaginal births after cesarean.

## Discussion

### Overview

The PowerMom platform is a dynamic research tool designed to capture and store maternal health data, enabling the analysis of longitudinal and episodic data across all stages of pregnancy. This includes survey responses, EHR, and wearable sensor data from pregnant and postpartum individuals. PowerMom’s ability to collect data from participants across all 50 US states, Puerto Rico, and Guam has facilitated the creation of a diverse dataset. The breadth and depth of these data allow for a detailed exploration of individual variations in pregnancies, helping researchers understand how these variations affect both maternal and fetal health. PowerMom’s data collection supports advanced analytical approaches, including trend analysis, comparative studies, and machine learning techniques. This enables the platform to explore patterns and associations between different maternal health factors, including demographic variables, health behaviors, and outcomes, offering insights that can inform personalized approaches to maternal care.

### Principal Findings

The PowerMom platform successfully engaged a diverse cohort of 5617 participants across all 50 US states, Puerto Rico, and Guam, demonstrating its effectiveness as a scalable tool for maternal health research. Of the 5617 participants, 3922 (69.8%) shared demographic data, revealing meaningful representation across age, racial and ethnic categories, and geographic regions. Nearly half (2723/5617, 48.5%) of participants were younger than 35 years, while 19.3% (1083/5617) were 35 years or older. Participants identifying as Black or African American accounted for 13.7% (770/5617) of the cohort, while Hispanic or Latina individuals represented 14.0% (788/5617). These data underscore the platform’s success in reaching historically underrepresented populations in maternal health research.

Recruitment efforts improved significantly over time, with enrollment increasing from 55 participants in late 2021 to 3310 in 2024. By 2024, the platform reached an average enrollment rate of 99.4 participants per week, reflecting the impact of enhanced recruitment strategies and partnerships with apps such as Philips Pregnancy+ and Mae Health.

Comprehensive data collection was achieved through surveys, wearable devices, and EHR integration. A total of 17,123 surveys were completed across the cohort. The initial Screener Survey had a completion rate of 100%. However, subsequent surveys saw a decline in completion rates: 71.8% (4033/5617) for the Intake Survey, 56.0% (3145/5617) for the Health History Survey, and 12.4% (697/5617) for the Postpartum Survey. These trends highlight the need for strategies to sustain participant engagement over time.

Wearable device data were provided by 1168 participants, yielding more than 378,000 daily biometric measurements, including activity levels, sleep duration, and resting heart rate. These continuous data provide a granular, real-time view of maternal health throughout pregnancy and postpartum stages. In addition, 96 participants connected their EHRs to the platform, contributing 276 distinct data points, such as diagnoses, medications, and laboratory results. The rationale for the low EHR connection rate was not a metric of our study. However, we observed that the low willingness to share EHR data among participants (1.7%) is consistent with findings in the literature, such as the study by Kim et al [30]. Key factors cited in that study include privacy concerns, the lack of trust in data-sharing platforms, and technical challenges in linking EHRs. In addition, the demographic diversity within the cohort may have contributed to this hesitancy, as some populations with historical mistrust of health care systems might have been more hesitant to share sensitive information. Understanding these barriers is critical for future studies aiming to integrate EHR data more comprehensively.

The analysis of pregnancy and delivery-related characteristics further enriched the dataset. Approximately 28.1% (1578/5617) of participants enrolled during the first trimester, and 15.1% (849/5617) completed their pregnancies during the study period. Delivery data revealed that 9.1% (512/5617) of participants reported experiencing spontaneous vaginal births, while 2.1% (117/5617) and 2.9% (163/5617) underwent planned and unplanned cesarean deliveries, respectively. Mental health data indicated that 14.9% of participants reported receiving treatment for anxiety or depression at baseline.

Despite the platform’s successes, notable data gaps were observed, including missing demographic information (eg, age data missing for 1811/5617, 32.2% of participants) and survey attrition. Due to attrition, only 15.1% (849/5617) of participants reported completing their pregnancies within the study period, limiting delivery-related analyses. These findings highlight both the potential and challenges of using digital platforms for maternal health research.

### Comparison With Previous Work

The PowerMom platform builds upon lessons learned from our earlier digital pregnancy study, the Healthy Pregnancy Study [31]. The Healthy Pregnancy Study was embedded within a pregnancy app, allowing for seamless recruitment and the enrollment of more than 4000 participants. The study generated more than 14,000 individual surveys and more than 107,000 daily health measurements, including sleep, activity, and heart rate. The Healthy Pregnancy Study also highlighted key disparities in maternal health care access and treatment. For example, Black and rural participants reported lower use of prenatal vitamins, antiemetics, and antidepressants compared with their non-Black and urban counterparts [32]. PowerMom sought to build on these findings by enabling widespread participation from underrepresented communities and offering valuable insights into how structural and health care factors affect maternal health outcomes.

### Challenges Encountered

#### Substudy Enrollment Challenges

PowerMom’s foundational protocol allows for the integration of additional substudies targeting specific research questions. However, when substudies have additional eligibility criteria, ensuring that participants can easily meet those criteria can be challenging. For example, incomplete baseline surveys or unmet eligibility criteria can hinder participant enrollment in substudies. Streamlining the baseline survey process and providing clear prompts for participants to join substudies have proven to be effective strategies in minimizing these challenges and boost substudy engagement.

#### Fraudulent Activity

One unexpected challenge during one of the PowerMom substudies was the fraudulent redemption of gift cards offered as participation incentives. The platform’s automated system was exploited by 580 participants who enrolled fraudulently to claim gift cards, leading to a rapid spike in enrollment in both the baseline study and PowerMom Connect. To counter this, we introduced anomaly detection measures, including monitoring the time taken to complete eConsent and flagging participants with irregular patterns of completion. These measures successfully curtailed fraudulent activity. Future studies should incorporate automated fraud detection tools from the outset to prevent disruptions.

#### Attrition in Survey Completion

Participant attrition in completing surveys over time remains a key limitation of the PowerMom platform. While initial survey completion rates were high, subsequent surveys saw lower completion rates, potentially due to participant fatigue or competing priorities. Enhanced engagement strategies, such as personalized incentives, interactive content, and dynamic reminders, are needed to reduce participant burden and improve survey retention in future studies.

### Limitations

While the PowerMom platform leverages decentralized and digital approaches to reach a large and diverse population, it is not without limitations. Despite the increasing penetration of smartphones in the United States (with 92% of adults owning a smartphone as of 2023 [33]), there remain disparities in broadband access and digital literacy, particularly in rural areas and among lower-income populations. These barriers may prevent certain individuals from participating fully in a purely digital, mobile-based platform. However, PowerMom’s decentralized design mitigates the need for participants to live near academic medical centers. Therefore, increasing access to maternal health research for those in remote or underserved areas.

In addition, as noted in the *Attrition in Survey Completion* section, participant attrition in survey completion remains a challenge for longitudinal studies such as PowerMom. Ensuring sustained engagement throughout pregnancy and postpartum periods is critical for comprehensive data collection.

### Lessons Learned

The relaunch of PowerMom in 2021 highlighted several key lessons for future digital health studies:

1. Scalability and adaptability: The platform’s scalability and adaptability have proven essential in accommodating diverse participant needs. Culturally tailored outreach strategies and bilingual interfaces were particularly successful in engaging historically underrepresented populations.
2. Continuous data collection: The ability to collect real-world, continuous data from wearables and EHR systems provides a comprehensive view of maternal health that surpasses traditional, episodic clinic visits. This real-time data collection allows for more precise tracking of health changes over time.
3. Fraud detection and prevention: The experience of dealing with fraudulent activity emphasized the importance of implementing robust anomaly detection systems to protect the integrity of participant data and incentives. Future studies should incorporate automated fraud detection tools from the outset to avoid disruptions.
4. Participant retention: Attrition in survey completion is a challenge in longitudinal digital health studies. Future studies should incorporate strategies to maintain participant engagement, such as personalized incentives, interactive content, or dynamic reminders, which may help reduce participant fatigue and improve data completeness.

### Conclusions

The PowerMom platform has demonstrated its potential as an innovative, scalable, and inclusive maternal health research tool. By leveraging decentralized, mobile-based recruitment and data collection, PowerMom successfully engaged a diverse cohort of 5617 participants across all 50 US states, Puerto Rico, and Guam. The platform achieved representation across critical demographic groups, including underrepresented racial and ethnic populations and individuals residing in areas with limited access to maternity care. Despite the platform’s successes, challenges such as survey attrition, missing data, and fraudulent activity underscored the importance of implementing robust engagement and data quality strategies in future digital health studies.

Looking ahead, PowerMom’s flexible and scalable architecture positions it as a transformative model for maternal health research. By continuing to enhance participant engagement strategies and expanding data collection capabilities to include genomic, environmental, and social determinants of health, PowerMom can generate actionable insights to address maternal health disparities. Ultimately, this platform represents a significant step toward improving maternal health outcomes, promoting health equity, and empowering diverse populations to actively participate in research that directly impacts their care.

## Appendix

Multimedia Appendix 1 Participant flow and in-app surveys from the PowerMom study, conducted between 2021 and 2024. This figure illustrates the step-by-step flow of participants from recruitment through study completion, including survey engagement. Surveys captured demographic, health history, and pregnancy-related data at various stages, leveraging the PowerMom app's user-friendly interface to enhance participant engagement and data collection.

## Abbreviations

ADI: Area Deprivation Index
eConsent: electronic informed consent
EHR: electronic health record
HIPAA: Health Insurance Portability and Accountability Act

## Data Availability

The datasets generated and analyzed during the PowerMom study are not publicly available due to privacy and confidentiality restrictions. The study collected sensitive health and demographic data from participants, which are deidentified but remain protected under institutional review board protocols. Researchers who wish to access the data for legitimate academic purposes can submit a formal request to PowerMom principal investigator, TA. Requests will be evaluated on a case-by-case basis to ensure compliance with ethical guidelines and data use agreements. The code used for data analysis is available upon request from the corresponding author.
