# Supplementary material for: Digital Health Platform for Maternal Health: Design, Recruitment Strategies, and Lessons Learned From the PowerMom Observational Cohort Study
Source: JMIR Form Res. 2025 Apr 7;9:e70149. doi: 10.2196/70149 (PMC12012398; doi:10.2196/70149)
Supplement: Multimedia Appendix 1 [file formative_v9i1e70149_app1.docx]

## APPENDIX

Appendix - Participant Flow

Appendix - Surveys
